# Antiseptic Drainage of the Lung

**Published:** 1889-11-09

**Authors:** 


### The Practitioner's Outlook and Retrospect.

[*The Editor will be glad to receive offers of co-operation and contributions from members of the profession. There is no doubt that much valuable material full of interest and instruction is at present lost to science. This is largely so in the case of* (1) *the younger and less-known members of the hospital staff,* (2) *those connected with cottage hospitals, and* (3) *the great body of medical practitioners not attached to any institution. The co-operation of all is cordially invited to enable the Editor to make* The Hospital *of the utmost possible use and value to the profession. Specialists willing to review books on their special subjects are invited to communicate this fact at once. All letters should be addressed to* The Editor, The Lodge, Porchester Square, London, W.]

### MEDICINE.

### ANTISEPTIC DRAINAGE OF THE LUNG.

Drs. Malagola and Montalti report a case of antiseptic drainage of the lung, in which symptoms of gangrene had supervened on severe intermittent fever. All treatment having failed, and the patient becoming daily worse, they introduced five centimetres (2in.) of a tube, two millimetres ($\frac{2}{10}$in.) in diameter, through an incision in the intercostal space below the right nipple. At first the cavity was washed out by means of the tube every three hours with a five per cent. solution of carbolic acid, but as it caused vomiting and violent cough, a solution of corrosive sublimate 1 in 2,000 was substituted. This was well borne, and the patient's symptoms began immediately to improve. After four days the tube showed a tendency to be expelled; it was gradually shortened, and on the nineteenth day withdrawn. The man's whole stay in hospital was 10 weeks, when he left quite well, with a marked depression of the chest wall anteriorly indicating the obliteration of the gangrenous cavity.

